# Antimetastatic Effect of Halichondramide, a Trisoxazole Macrolide from the Marine Sponge *Chondrosia corticata*, on Human Prostate Cancer Cells via Modulation of Epithelial-to-Mesenchymal Transition

**DOI:** 10.3390/md11072472

**Published:** 2013-07-15

**Authors:** Yoonho Shin, Gi Dae Kim, Ju-eun Jeon, Jongheon Shin, Sang Kook Lee

**Affiliations:** College of Pharmacy, Natural Products Research Institute, Seoul National University, Seoul 151-742, Korea; E-Mail: dicafree5@snu.ac.kr (Y.S.); gidaekim@snu.ac.kr (G.D.K.); mirabn@snu.ac.kr (J.J.); shinj@snu.ac.kr (J.S.)

**Keywords:** halichondramide (HCA), antimetastasis, PC3 cells, PRL-3, MMPs

## Abstract

Halichondramide (HCA), a trisoxazole-containing macrolide isolated from the marine sponge *Chondrosia corticata* has been shown to exhibit cytotoxicity and antifungal activities. In our previous study, HCA was also found to exhibit antiproliferative activity against a variety of cancer cells. However, the precise mechanism of action of HCA in the antitumor activity remains to be elucidated. In the present study, we identified the antimetastatic activity of HCA in the highly metastatic PC3 human prostate cancer cells. HCA showed potent growth inhibitory activity of the PC3 cells with an IC_50_ value of 0.81 µM. Further analysis revealed that HCA suppressed the expression of a potential metastatic biomarker, phosphatase of regenerating liver-3 (PRL-3), in PC3 cells. The suppression of PRL-3 by HCA sequentially down-regulates the expression of phosphoinositide 3-kinase (PI3K) subunits p85 and p110. The antimetastatic effect of HCA was also correlated with the down-regulation of matrix metalloproteases (MMPs) and the modulation of cadherin switches N-cadherin and E-cadherin. In addition, HCA also effectively suppressed the migration and invasion of PC3 cells. These findings suggest that halichondramide might serve as a potential inhibitor of tumor cell metastasis with the modulation of PRL-3.

## 1. Introduction

Natural products have served as important sources in drug discovery and development [1]. Although most of the current natural product-derived therapeutic drugs originated from terrestrial plant extracts marine-based natural products are recently considered to be an important resource for procurement of new chemical entities [2]. In particular, many compounds derived from marine sponges have exhibited anticancer activities with diverse mechanisms of action [3,4,5]. Eribulin mesylate, a structurally simplified macrolactone derivative of halichondrin B, which was originally isolated from the marine sponge *Halichondria okadai*, is an example of recently approved anticancer drug for metastatic breast cancer [6]. Moreover, in our previous study, we found that oxazole-containing macrolides from the marine sponge *Chondrosia corticata* exhibit potential cytotoxicity and antifungal activity [7]. Recently, we also reported the antiproliferative effect of (19*Z*)-halichondramide, a trisoxazole-containing macrolide from *C. corticata*, on human lung cancer cells via G2/M cell cycle arrest and suppression of mTOR signaling pathway [8]. However, the mechanism underlying the antimetastatic activity of trisoxazole-containing macrolides from *C. corticata* has not yet been elucidated.

Cancer metastasis is considered to be a major cause of cancer death. Indeed, the acquired increasing motility and invasiveness of cancer cells enhance the metastatic processes from the primary sites to secondary tissues [9]. Many distinctive biomarkers are eventually involved in each step of metastasis.

The phosphatase of regenerating liver (PRL) represents a novel subfamily of protein tyrosine phosphotases (PTPs); this subfamily contains three members (PRL-1, PRL-2, and PRL-3) that share a high degree (75%) of amino acid sequence identity [10,11,12]. In particular, PRL-3 has recently attracted a great deal of attention because of its association with tumor metastasis [13,14]. Elevated PRL-3 mRNA levels have been found in many cancer cells including colon, lung, and prostate. In addition, PRL-3 overexpression was found in nearly all metastatic lesions that are derived from colorectal cancers [15,16]. Recent findings also suggested that overexpression of PRL-3 promotes motility and metastasis of mouse melanoma cells both *in vitro* cell culture and *in vivo* mouse model [17,18]. These data might provide PRL-3 as a novel biomarker in the association of the metastatic properties of tumor cells. However, little is known about the underlying mechanisms by which PRL-3 promotes cell invasion and growth.

In this study, we report for the first time that halichondramide, a trisoxazole-containing macrolide isolated from *C. corticata*, exhibits potent antimetastatic activity in human prostate cancer cells that is associated with the modulation of both PRL-3 and various metastasis biomarkers.

## 2. Results

### 2.1. Growth Inhibitory Activity of Halichondramide in PC3 Prostate Cancer Cells

**Figure 1:**
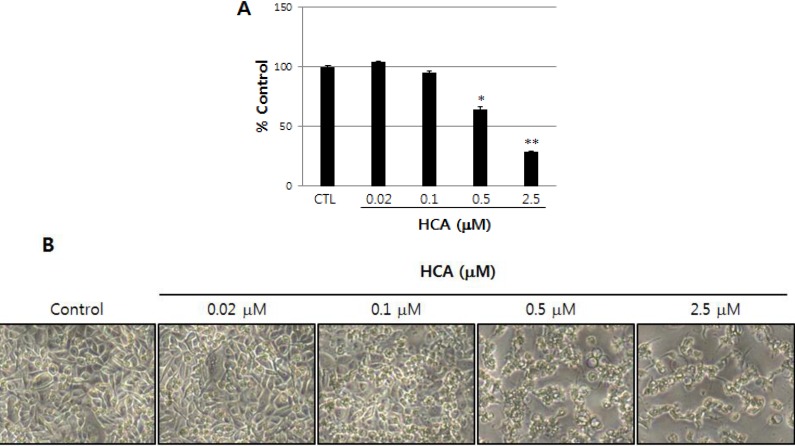
The growth inhibitory activity of halichondramide (HCA) in PC3 prostate cancer cells. (**A**) PC3 cells (1 × 10^5^ cells/mL) were treated with HCA for 72 h; subsequently, antiproliferative activity was determined by the SRB protein dye method as described in the Experimental Section. The data are represented as the mean percentages ± SD for each HCA-treated group relative to the DMSO-treated control group.Each experiment was performed in triplicate (*n =* 3). * *p* < 0.05, ** *p* < 0.01 compared with the control group; (**B**) The morphological changes of PC3 cells treated with HCA for 72 h were observed under a phase-contrast microscope and photographed (at 40×magnification).

Antiproliferative potential of halichondramide (HCA) in PC3 human prostate cancer cells was evaluated using the sulforhodamine B (SRB) assay. As illustrated in Figure 1A, HCA exhibited antiproliferative activity against PC3 cells in a concentration-dependent manner, and the IC_50_ value was 0.81 µM for an incubation of 72 h. In particular, the highest concentration (2.5 µM) of HCA produced a remarkable decrease in cell numbers (Figure 1B), suggesting that HCA might exhibit a cytostatic effect at relatively low concentrations but exert a cytotoxic effect at higher concentrations.

### 2.2. Modulation of the Gene Expression Levels of PRL-3, MMPs, and Cadherins by HCA

**Figure 2:**
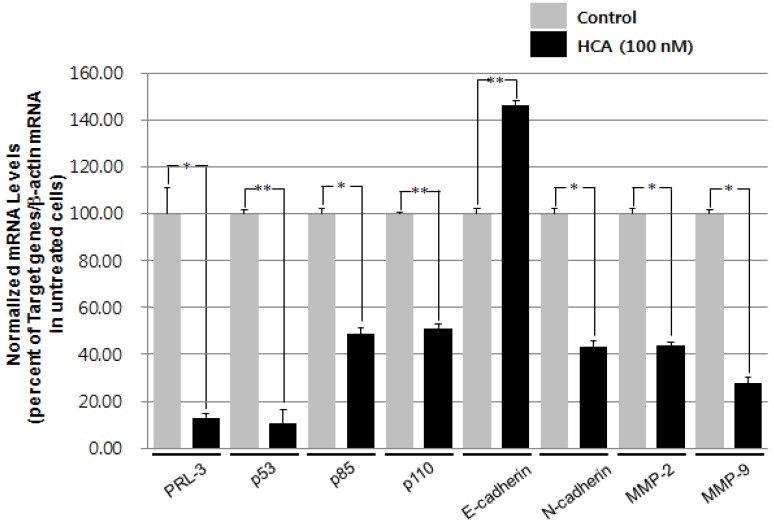
The effects of HCA on the mRNA expression of PRL-3 and its down-stream genes and metastatic biomarkers. PC-3 cells (4 × 10^5^ cells/mL) were treated with HCA for 24 h, and the mRNA expression levels of PRL-3 and metastasis biomarkers were determined by real-time RT-PCR analysis. The data represent the mean values ± SD of nine experiments. * *p* < 0.05, ** *p* < 0.01 compared with the control group.

The modulation of epithelial-mesenchymal transition (EMT) is regarded as an important target in metastatic regulation. To evaluate whether HCA is able to suppress the metastatic potential of highly metastatic cancer cells, the modulation of the expression levels of genes that are associated with metastasis was examined using real-time RT-PCR analysis. HCA significantly down-regulated the mRNA expressions of matrix metalloproteinase-2 (MMP2), MMP9, and N-cadherin, whereas the expression of E-cadherin was up-regulated after treatment of HCA for 24 h (Figure 2). In addition, HCA suppressed the expression of PRL-3, a metastasis-associated marker, and of the PI3 kinase subunits p85 and p110, which are downstream targets of PRL-3. These results suggest that HCA is a potential inhibitor of EMT in human adenocarcinoma prostate cancer cells via the modulation of PRL-3 and its downstream targets including PI3 kinase.

### 2.3. Suppressive Expression of PRL-3 and Its Associated Proteins by HCA

**Figure 3:**
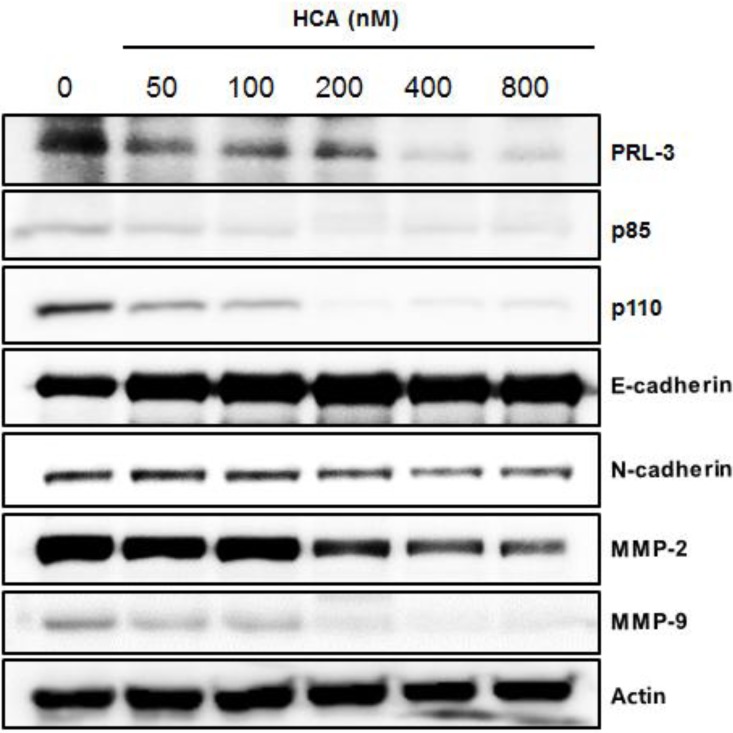
The effects of HCA on the expression of PRL-3 and its associated proteins. PC-3 cells (2 × 10^5^ cells/mL) were treated with HCA for 24 h; subsequently, the protein expression levels of PRL-3 and its associated proteins were analyzed by Western blotting as described in the Experimental Section.

To further clarify the mechanisms of action underlying the antimetastatic activity of HCA, signaling proteins that are overexpressed or inactivated in PC3 cells were examined by Western blot analysis. These analyses revealed that HCA treatment resulted in the down-regulation of the PRL-3 protein, which is known to be overexpressed in many metastatic cancer cell lines [15,19]; these findings are consistent with the suppression of the mRNA expression of PRL-3 by HCA. The pivotal downstream effector proteins of PRL-3, p85 and p110 were also suppressed by HCA (Figure 3). We further examined the effect of HCA on metastasis-associated protein expression levels and determined that the expression levels of MMP2 and MMP9 were significantly suppressed by HCA. In addition, examinations of E-cadherin and N-cadherin, additional cancer metastasis biomarkers that are known to act as a switch in epithelial-mesenchymal transition [20], revealed the up-regulation of E-cadherin but the down-regulation of N-cadherin following HCA treatment, further supporting the antimetasitatic effects of HCA (Figure 3). These data suggest that the antimetastatic activity of HCA is associated with the modulation of PRL-3, PI3 kinase, and EMT-related proteins in PC3 cells.

### 2.4. Inhibitory Effect of HCA on Cell Migration and Invasion

**Figure 4:**
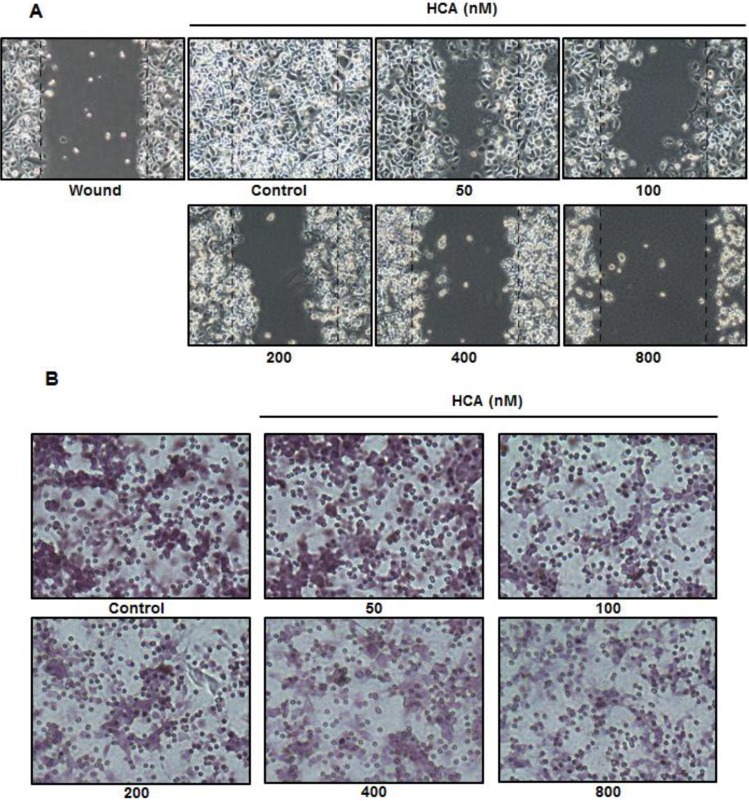
The inhibitory effects of HCA on cell migration and invasion. (**A**) The effect of HCA on cell migration was analyzed by the wound healing assay. PC3 cells (1×10^5^ cells/mL) were seeded in six-well plates and incubated for 48 h.An artificial wound was made that consisted of a scratch with a micropipette tip. The wound healing process was monitored during 72 h of incubation with various concentrations of HCA. Images of the wounds were photographed at 0 and 72 h under an inverted microscop; (**B**)The cell invasion assay was performed in a Matrigel-coated chamber system as described in the Experimental Section. PC3 cells (1 × 10^5^ cells/chamber) were plated in the upper chambers of Matrigel-coated Transwell insert. The lower chambercontained 0.1 mg/mL BSA as a chemoattractant in the medium. The inserts were incubated for 24 h; subsequently, the cells that had invaded the outer surface of the membrane were fixed, stained, and photographed.

Cellular migration and invasiveness are fundamental features of cancer metastasis. Given the suppressive effects of HCA on the expression levels of genes that are associated with metastasis, as depicted in Figure 3, further investigations were initiated to confirm the antimetastatic potential of HCA with respect to cellular migration and invasion. Wound healing and invasion assays were performed to evaluate the inhibitory effect of HCA on the migratory features of PC3 cells. In the wound healing assay, a wound scratch in vehicle-treated control cells was almost completely closed after 72 h of incubation. However, treatment with HCA resulted in the suppression of wound healing in a concentration-dependent manner (Figure 4A). In the invasion assay, HCA also demonstrated concentration-dependent inhibition of the invasion of PC3 cells through the embedded inner chamber (Figure 4B).

### 2.5. Suppressive Effect of HCA on the MMPs in Gelatin Zymography

**Figure 5:**
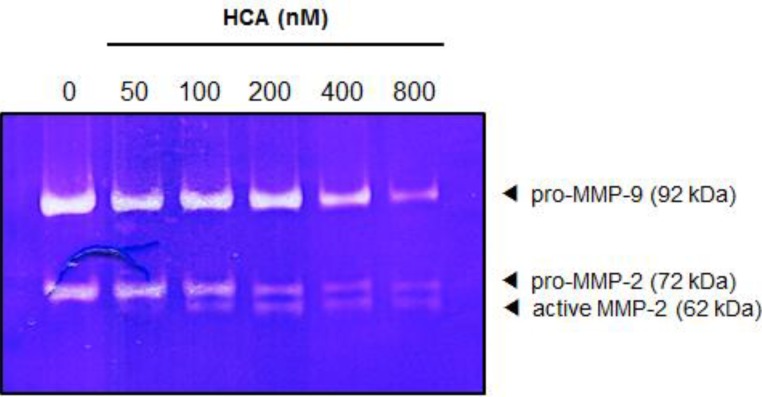
The inhibitory effects of HCA on gelatin degradation. The effect of HCA on the MMPs expression were also analyzed by the gelatin zymographic method. HT-1080 cells (5 × 10^4^ cells/mL) were treated with HCA for 72 h; subsequently, as described in the Experimental Section, the proteolytic activities of the MMPswere assessed by the degradation of gelatin.

To further determine whether HCA might affect the expression of MMP2 and MMP9, gelatin zymographic analysis was performed in HT-1080 human fibrosarcoma cells. The cells (5 × 10^4^ cells/mL) were treated with HCA for 72 h; following this treatment, protein lysates were resolved on a 0.2% gelatin-containing SDS-polyacrylamide gel. The degradation of the gelatin substrate by the MMP2 and MMP9 was clearly detected in untreated control cells, whereas treatment with HCA markedly inhibited the expressions of MMP2 and MMP9 in a concentration-dependent manner (Figure 5). This result is well correlated with the down-regulation of MMP-2 and MMP-9 protein expression by HCA in PC3 cells (Figure 3).

## 3. Discussion

The metastasis of cancer cells is an important factor in most deaths of cancer patients [21]. The increase in the motility and invasiveness of cancer cells has also been found to be associated with an enhanced tendency to undergo epithelial-mesenchymal transition (EMT) [22]. In particular, functional cadherins are highly associated with the progression of EMT. For example, the up-regulation of N-cadherin induces invasiveness of tumor cells, whereas the loss of E-cadherin also enhances the destruction of cell-cell adhesion junctions, and thus these events might be considered to be the important contributors of EMT [22].

In this study, we demonstrated the antiproliferative and antimetastatic activities of halichondramide (HCA) in human prostate cancer cells. The antiproliferative activities of trisoxazole-containing macrolides, including HCA against a variety of human cancer cell lines, such as lung (A549), colon (HCT116), breast (MDA-MB-231), liver (SK-HEP-1), and stomach (SNU601) cancers, have recently been reported by our group [8]. In agreement with this previous result, HCA exhibited antiproliferative activity against the highly metastatic human PC3 prostate cancer cells. Although oxazole-containing macrolides have exhibited antiproliferative activity against cancer cells [8,23], the antimetastatic effects of these classes of compounds on cancer cells have not previously been reported. Therefore, we further determined the antimetastatic potential of HCA using highly metastatic prostate cancer cells. HCA modulated the expression of various biomarkers that are associated with metastasis, including PRL-3, p85, p110, E-cadherin, N-cadherin, MMP2, and MMP9, at both the transcriptional and translational levels. Wound healing and Matrigel invasion assays revealed that HCA also inhibited cellular migration and invasion. Recent studies have demonstrated that phosphatase of regenerating liver-3 (PRL-3) is a potential biomarker of cancer metastasis [24]. PRL-3 was first identified as a protein that could play an essential role in cancer metastasis in a genome-wide transcriptional analysis of colorectal cancer samples [19]. Subsequent studies have found that the overexpression of PRL-3 is highly correlated with increased metastasis in human cancers [15]. This finding suggests that PRL-3 might be a potential target for human cancer therapy, particularly, with respect to cancer metastasis. In the present study, the overexpression of PRL-3 was detected in PC3 cells, and HCA significantly suppressed the expression of PRL-3 at both transcriptional and translational levels in these cells. The expression of subunits p85 and p110 of PI3K, one probable downstream target of PRL-3, was also down-regulated by HCA. These results demonstrate the involvement of PRL-3 in metastasis and the potential of HCA for producing antimetastatic effects in cancer cells. In addition, the analysis of EMT biomarkers is indicative of the metastatic characteristics of cancer cells. An examination of cadherins revealed that N-cadherin was highly overexpressed in PC3 cells but E-cadherin, a counterpart of N-cadherin, was maintained at low levels in these cells. However, treatment with HCA suppressed the expression of N-cadherin but activated the expression of E-cadherin in PC3 cells. These results additionally confirmed the antimetastatic potential of HCA in PC3 cells. The suppression of the matrix metalloproteinases MMP2 and MMP9 and the inhibition of the enzymatic activities of these metalloproteinases further confirmed the antimetastatic and antiinvasive activities of HCA in highly metastatic cancer cells. PRL-3 has been shown to promote the proliferation of various cell lines [25,26,27]. The phosphatidylinositol-3-kinase/protein kinase B (PI3K/AKT) signaling pathway is also an important driver of cell proliferation and survival [28]. Our findings indicate that HCA down-regulates the expression of PRL-3 and its downstream target PI3K, abrogating PRL-3-mediated AKT activation. This process might represent one plausible mechanism of action underlying the antimetastatic and antiproliferative activity of HCA in human prostate cancer cells. Although we found the antimetastatic activity of HCA with the modulation of several biomarkers associated with the metastasis the precise mechanism of action of HCA is still unclear and further detailed studies are needed for the anticancer activity for prostate cancer cells. Recent metabolomics studies gave important information for the progression of aggressive prostate cancer phenotype by androgen receptor activation [29,30]. Therefore, this approach might be also applicable to elucidate the further detailed mechanism of action of HCA for prostate cancer cells.

## 4. Experimental Section

### 4.1. Reagents

**Figure 6:**
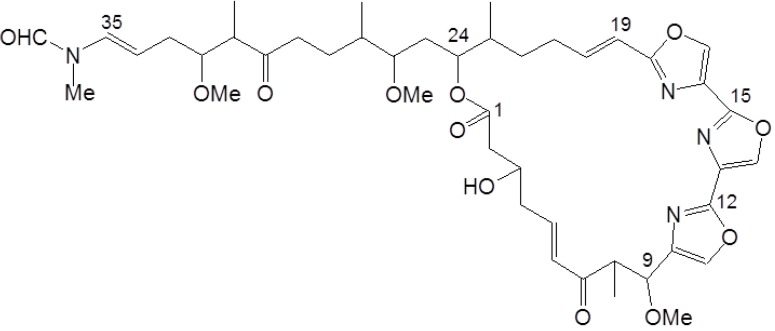
Chemical structure of halichondramide.

Dimethyl sulfoxide (DMSO), bicinchoninic acid (BCA), copper sulfate, trichloroacetic acid (TCA), and thiazolyl blue tetrazolium bromide (MTT) were purchased from Sigma-Aldrich (St. Louis, MO, USA). Roswell Park Memorial Institute (RPMI) 1640 medium, fetal bovine serum (FBS), trypsin-EDTA solution (1×), and antibiotic-antimycotic solution (100×) were purchased from Invitrogen (Carlsbad, CA, USA). Mouse monoclonal anti-E-cadherin and anti-N-cadherin were purchased from BD Biosciences (San Diego, CA, USA). Rabbit polyclonal anti-p85, anti-p110, anti-MMP2, and anti-MMP9 were purchased from Cell Signaling Biotechnology (Danvers, MA, USA). Mouse monoclonal anti-PRL-3 was purchased from Santa Cruz Biotechnology (Santa Cruz, CA, USA). The test compound halichondramide (HCA), a trisoxazole-containing macrolide (Figure 6), was isolated from the marine sponge *Chondrosia corticata* in accordance with previously described methods [7] and dissolved in 100% dimethyl sulfoxide (DMSO).

### 4.2. Cell Culture

Human prostate adenocarcinoma PC3 cells were obtained from the American Type Culture Collection (Manassas, VA, USA), and were cultured in RPMI-1640 medium supplemented with 10% heat-inactivated fetal bovine serum, 100 units/mL penicillin, 100 μg/mL streptomycin, and 250 ng/mL amphotericin B at 37 °C in a humidified incubator under an atmosphere containing 5% CO_2_.

### 4.3. The Evaluation of Antiproliferation Activity

Cell proliferation was measured using the sulforhodamine B (SRB) assay [31]. Briefly, PC3 cells (4 × 10^5^ cells/mL) were seeded in 96-well plates with various concentrations of HCA and incubated at 37 °C in a humidified atmosphere with 5% CO_2_. After 72 h of HCA treatment, the cells were fixed with a 10% TCA solution for 1 h, and cellular proteins were stained with 0.4% SRB in a 1% acetic acid solution. The stained cells were dissolved in 10 mM Tris buffer (pH 10.0). The effect of HCA on cell proliferation was calculated as a percentage, relative to a solvent-treated control, and the IC_50_ values were determined using nonlinear regression analysis (percent survival *versus* concentration).

### 4.4. Analysis of Gene Expression by Real-Time RT-PCR

Real-time RT-PCR was employed to determine the gene expression of PRL-3 in PC3 cells. Briefly, PC3 cells (2 × 10^5^ cells/mL) were cultured in 100 mm dishes for 24 h. The cells were treated with HCA for an additional 24 h. Total cellular RNA was extracted with TRIzol reagent and reverse transcribed at 42 °C for 60 min with 0.5 μg of oligo(dT)15 primer in a reaction volume of 20 μL, using reverse transcription system (Promega, MI, USA). Specific gene primers were designed and were subsequently custom synthesized by Bioneer Corporation (Daejon, Korea). The primer sequences that were used in this study are listed in Table 1. Real-time PCR was conducted using a MiniOpticon system (Bio-Rad, Hercules, CA, USA); each PCR amplification included 5 μL of reverse transcription product, iQ SYBR Green Supermix (Bio-Rad, Hercules, CA, USA), and primers in a total volume of 20 μL. The following standard thermo cycler conditions were employed: 95 °C for 20 s prior to the first cycle; 40 cycles of 95 °C for 20 s, 56 °C for 20 s, and 72 °C for 30 s; 95 °C for 1 min; and 55 °C for 1 min. The threshold cycle (C_T_), indicating the fractional cycle number at which the amount of amplified target gene reaches a fixed threshold for each well, was determined using the MJ Opticon Monitor software package (Bio-Rad, Hercules, CA, USA). Relative quantification, representing the change in gene expression in real-time quantitative PCR experiments between a sample-treated group and the untreated control group, was calculated by the comparative C_T_ method in accordance with previously described methods [32]. The data were analyzed by evaluating the expression 2^−ΔΔC_T_^, where ΔΔC_T_ = [C_T_ of target gene − C_T_ of housekeeping gene]treated group − [C_T_ of target gene − C_T_ of housekeeping gene]untreated control group. For the treated samples, the evaluation of 2^−ΔΔC_T_^ represents the fold change in gene expression relative to the untreated control, normalized to a housekeeping gene (β-actin). Real-time PCR primer sequences are listed in Table 1.

**Table 1:** The sequences of the primer pairs that were used to examine specific target genes in RT-PCR analysis.

Target gene	Sequences	
PRL-3	Sense	5′-AGA AGT ACG GGG CTA CCA CTG-3′
	Antisense	5′-CAC AAC GGT GAT GCC ATC CTT-3′
p85	Sense	5′-CTG CCT CCT AAA CCA CCA AAA-3′
	Antisense	5′-TTC ATA CCG TTG TTG GCT ACA G-3′
p110	Sense	5′-CCA CGA CCA TCA TCA GGT GAA-3′
	Antisense	5′-CCT CAC GGA GGC ATT CTA AAG T-3′
E-cadherin	Sense	5′-CGA GAG CTA CAC GTT CAC GG-3′
	Antisense	5′-GGG TGT CGA GGG AAA AAT AGG-3′
N-cadherin	Sense	5′-AGC CAA CCT TAA CTG AGG AGT-3′
	Antisense	5′-GGC AAG TTG ATT GGA GGG ATG-3′
β-Actin	Sense	5′-AGC ACA ATG AAG ATC AAG AT-3′
	Antisense	5′-TGT AAC GCA ACT AAG TCA TA-3′
MMP2	Sense	5′-GAT ACC CCT TTG ACG GTA AGG A-3′
	Antisense	5′-CCT TCT CCC AAG GTC CAT AGC-3′
MMP9	Sense	5′-TGT ACC GCT ATG GTT ACA CTC G-3′
	Antisense	5′-GGC AGG GAC AGT TGC TTC T-3′

### 4.5. Western Blot Analysis

Cells were treated with the test compound with various concentrations for 24 h. Each sample was homogenized in ice-cold 1× ProPrep buffer (iNtRON Biotechnology, Seoul, Korea) and incubated for 20 min on ice. The lysates were centrifuged at 12,000× *g* for 20 min, and the supernatant was collected and stored at −80 °C. Total protein contents were determined using the BCA method. Proteins (30 μg) were denatured by boiling in a lysis buffer (250 mM Tris-HCl at pH 6.8, 4% SDS, 10% glycerol, 0.006% bromophenol blue, 2% β-mercaptoethanol and 2 mM sodium ortho-vanadate) for 5 min. The same amount of protein in each lysate was loaded and separated by SDS–polyacrylamide gel electrophoresis and then electrotransferred to a PVDF membrane. Nonspecific binding was blocked with 5% BSA in TBS; subsequently, the membranes were incubated with primary antibodies diluted in TBS-Tween 20 containing 3% BSA (1:200–1:1000) overnight at 4 °C, washed three times with TBS-Tween 20, and incubated with corresponding HRP-conjugated secondary antibodies. The protein was detected using a LAS-1000 Imager (Fuji Film Corp., Tokyo, Japan).

### 4.6. Wound Healing Assay

To assess the motility of cells exposed to the test compound, a wound healing assay was performed [33]. PC3 cells were seeded in six-well plates at a density of 1 × 10^5^ cells/mL and incubated for 48 h until they reached 80%–90% confluency. A confluent monolayer of PC3 cells was artificially wounded with a micropipette tip, and the detached cells were washed with serum-free RPMI1640 and treated with test compounds in growing medium for 72 h. The cells were washed twice with phosphate-buffered saline. Images of the wounds were photographed at 0 and 72 h under an inverted microscope (CKX41, Olympus).

### 4.7. Cell Migration Assay

PC3 cells (1 × 10^5^ cells/chamber) were used for migration assays as described previously [34]. Cells were seeded into the top chambers of a 24-well Matrigel-coated polyethylene terephthalate membrane inserts with 8 µm pores (Millipore, Billerica, MA, USA). The plates were coated with 10 μL of type I collagen (0.5 mg/mL) and 20 μL of a 1:2 mixture of Matrigel:RPMI1640. Cells were plated in the upper chamber of the Matrigel-coated Transwell insert. The medium of the lower chambers also contained 0.1 mg/mL bovine serum albumin as a chemoattractant. The inserts were incubated for 24 h at 37 °C. The cells that had invaded the outer surface of the membrane were fixed with methanol, stained with hematoxylin and eosin, and photographed.

### 4.8. Gelatin Zymography

HT-1080 cells were treated with various concentrations of HCA. After 72 h of incubation, conditioned media were collected, and gelatin zymographic analysis was performed to measure the expressions of MMP2 and MMP9 [35]. Briefly, the protein lysates extracted from the conditioned media were denatured by mixing 5× gel loading buffer containing 0.1 M Tris (pH 6.8), 50% glycerol, 2% SDS, and 0.1% bromophenol and electrophoretically separated on a polyacrylamide gel containing 0.2% gelatin. The resolved proteins in the gel were washed and renatured by the exchange of SDS with nonionic detergent Triton X-100 contained in washing buffer (50 mM Tris–HCl, pH 7.5, 100 mM NaCl and 2.5% Triton X-100), and further incubated with incubation buffer (50 mM Tris-HCl at pH 7.5, 150 mM NaCl, 10 mM CaCl_2_, 0.02% NaN_3_, and 1 μM ZnCl_2_) for 24 h at 37 °C with shaking. The incubated gel was stained with Coomassie Blue R-250, and the proteolytic activities of the MMPs were detected against a blue background as clear bands that resulted from the degradation of gelatin.

### 4.9. Statistics

The data are expressed as means ± S.D. The statistical analysis of the data was performed using SigmaStat 2.03 (SPSS, Chicago, IL, USA). One-way analysis of variance (ANOVA) was used to compare the mean responses among the treatments. A statistical probability of *P* < 0.05 was considered significant.

## 5. Conclusions

In summary, the present study demonstrates the potential antimetastatic and antiinvasive activity of halichondramide (HCA), a trisoxazole-containing macrolide from the marine sponge *Chondrosia corticata*, in human prostate cancer cells. This investigation provides the first plausible mechanism of action for the antimetastatic activity of HCA; this mechanism appears to involve the suppression of PRL-3 and its downstream signaling pathway, as evidenced by the HCA-related modulation of EMT biomarkers in prostate cancer cells. Because no appropriate therapeutic approach currently exists to treat prostate cancer that has recurred after surgery and/or radiation, these findings suggest that halichondramide might be a promising new chemical candidate for the management of human prostate cancer. This compound merits prioritization for additional pre-clinical and clinical studies that could provide a rationale for the development of halichondramide as a chemotherapeutic agent for prostate cancer.
